# Metropolitan Hospital Sunday Fund

**Published:** 1910-12-24

**Authors:** 


### METROPOLITAN HOSPITAL SUNDAY FUND.

### ANNUAL MEETING OF CONSTITUENTS.—IMPORTANT DISCUSSION.

The annual meeting of the constituents of this Fund was held at the Mansion House on Friday, December 16, under the presidency of the Right Hon. the Lord Mayor (Sir Thomas Vezey Strong), who is President and Treasurer. There was a good attendance, including Lord Cheylesmore, Sir William Church, Sir John Bell, the Chief Rabbi, Sir Edward Durning Lawrence, Sir Savile Crossley, and Sir Joseph Dimsdale.

Alderman Sir John Bell moved: "That the report of the Council for the year 1910 be, and is hereby, received." In doing so, he said that the report had been prepared at great length by the Committees, and the meeting would wish to express its gratification at the result of the collections for the year. The fact that the amount was slightly less than in the previous year was easily accounted for by the fact that there were no legacies received during that year. As a member of the Distribution Committee, he was able to say that the greatest care had been exercised so that a fair, equitable, and proper distribution of that large Fund, which did so much in the interests of a great community, should be arrived at. Of course every gratitude was felt to the clergy of all denominations for what they have done; but doubtless a separate resolution would deal with that. But he wished to express his personal gratification that the Committees had increased the amount allotted for surgical appliances from 5 per cent. to 7½ per cent., which decision the present meeting would be asked to endorse. No one knew more than did the members of the Committees of that great institution how much good was done by the agencies for supplying surgical apparatus, and he was sure the meeting would wish to give as much financial help as possible to those institutions.

### The Sunday Fund and Medical Education.

The resolution having been seconded, the Rev. L. S. Lewis said there were three questions he would like to ask before the resolution was put. The first was: Why was the grant to St. George's Hospital increased from £198 in 1909 to £1,545 in 1910? Had there been any extra calls upon the hospital, and, if so, of what nature? The second question was: Was the statement that St. George's Hospital had transferred £1,576—almost the same sum as the grant—from the General Fund of the hospital to its medical school brought before the Distribution Committee? The third question was: Was the Council, in recommending that grant to the meeting, aware that the King Edward's Hospital Fund had suspended its grant to St.

George's Hospital this year until it was satisfied with the financial relations between the hospital and the medical school?

The Chairman asked Sir William Church, Chairman of the Distribution Committee, to reply to the questions.

Sir William Church said the award to St. George's Hospital was made upon exactly the same terms as to all the other hospitals. Upon the accounts which were returned to the Sunday Fund, what was called a basis in accordance with the expenditure of each hospital upon the maintenance of its patients was formed, and then each hospital got a proportion of its share in accordance with what were the figures of its basis. Why St. George's Hospital had so small a grant last year as compared with this year was simply that that hospital had very large legacies in the preceding year, which placed their accounts entirely upon the right side of the ledger. With regard to the second question, all he could say was that no unusual steps were taken by the Distribution Committee. The basis of the hospital was arrived at in the same way, and the Committee accepted the statement of the hospital that they had a certain amount of money which was called the Discretionary Fund.

The Chairman pointed out that the third question had not been answered, to which Sir William replied that he did not feel called upon to answer that. The grant from the Fund was made in August, before any difficulty arose in regard to the King's Fund.

The Rev. L. S. Lewis said that, in view of those answers, he would like to move an amendment to the motion that the report be adopted. His amendment was: "That the report be adopted, with the exception of the grant of £1,545 15s. 10d. to St. George's Hospital, which shall be referred back to the Distribution Committee for further consideration; and that that Committee shall report to a general meeting called for the purpose when King Edward's Hospital Fund has publicly declared itself satisfied with the financial relations between that hospital and the medical school." Mr. Lewis said he thought it would be in the knowledge of every one present that for some years there had been a very strong feeling on the part of a portion of the public at least, that funds which were given for the relief of the sick poor had gone to medical schools. In 1904 the matter reached a head, and the result of the agitation was that a Commission was appointed by the King Edward Hospital Fund—consisting of Mr. Justice Fry, Lord Welby, and the present Archbishop of York—to inquire into the statements which were made. That Commission found that the existence of a hospital school was a great help to the hospital. They also found that the school derived equal benefits from the hospital, and that those two institutions were a complete set-off against the other. Therefore they said that anything which might be taken from the hospital funds for the school should be regarded as in the nature of a debt from the school to the hospital. That Commission also made a very clear finding, and made the following recommendation: "We venture to submit that the distinction between the hospital and the school should in every case be drawn, not only definitely and exactly, but with such clearness that it may be understood by the general public, and so that no question may arise as to the discretion and application of moneys contributed, whether from the King's Fund or from any other source." The result of that Commission was that a certain amount of peace was arrived at. That peace had now been disturbed by the action of St. George's Hospital. In 1908 a distinguished member of its staff made a speech in which he referred to the ill-advised action of that Commission. That was followed by an open avowal that henceforth the usual payments to the medical school for work done for the hospital, which had previously been paid from the Discretionary Fund, were now paid out of the General Fund of the hospital.

The Chairman, answering an interruption on a point of order by a member of the Council, said he did not think Mr. Lewis realised that King Edward's Fund was a separate authority, and what he was quoting might not appeal to, or be regarded as binding on, the present Fund.

The Rev. Mr. Lewis, resuming, said he agreed, but wished to show that St. George's Hospital had deliberately said that henceforth they would pay out of the General Fund of the hospital money to the school. ("No, no.")

The Chairman: I think that is a mistake. It was clearly stated that St. George's Hospital had a discretionary power. Their subscribers said they would give a guinea to Fund A for the Hospital, or to Fund B, the Discretionary Fund, out of which it was lawful for the Board of Management to contribute a portion, if they so willed, to the medical school. (Hear, hear.) It was only out of that Discretionary Fund that they supported the school. (Hear, hear.)

Mr. Lewis rejoined that if he had thought that was the case he would not have brought the matter forward. But on page 4 of the 1909 Report appeared the following words: "The usual payments to the medical school for work done for the hospital, which have previously been paid by the Discretionary Fund, are now paid from the General Fund Account." ("It is nothing to do with this Fund, nothing to do with us at all.")

The Chairman assured Mr. Lewis that the meeting did not wish to refuse him a hearing, but those at that meeting could not interfere with the conduct of St. George's Hospital in carrying out its work.

Mr. Lewis assured the meeting that he did not intend to suggest that St. George's should be dictated to by the Hospital Sunday Fund, but his object was to show that at the present time King Edward's Fund, which appointed the Commission he had referred to, was not satisfied with the financial relations between that hospital and its school. That being so, his opinion was that at such a moment as this it would be wrong, and, in his view, nothing short of a scandal, if, without question, such a statement as that £1,500 had been given away from the hospital funds to the school should be allowed to pass, and that, while King Edward's Fund was dissatisfied, this Fund should give the same sum. He scarcely saw how anybody could object to the first part of his motion, though people might reasonably argue on the remainder. If one Fund were to work against another like that it would place every hospital above the control of the Funds, and make them independent. In regard to the second part of the motion, he felt there was a certain difficulty. The result of his amendment, if carried, would be that St. George's Hospital would not receive any grant at all, that the Sunday Fund grant would fail unless the King Edward Hospital Fund was satisfied.

The Chairman: The grant was given to them last August, and they have probably spent it long before this.

Mr. Lewis said that remark threw an interesting sidelight on the Constitution, which made him sorry that he did not press his last year's resolution dealing with the constituents having a voice in the management of the Funds.

The Chairman pointed out that the constituents chose certain Committees to do the work of the Fund, consisting of gentlemen in whom they had complete confidence, and who, in the exercise of such confidence, had done certain things, and they came to the meeting constitutionally to ask the constituents to approve of their action. That was the question now before the meeting, whether it would approve what the Council had done in its name. (Applause.)

Mr. Lewis said it was admitted that the money had been granted, and as that could not be recalled the constituents sat there like so many dolls. Surely they could have a voice in what was done, even if it was a voice and nothing else. He could not understand any Minister asking for contributions to a Fund over which they had no control. He did not suggest that this Fund should be ruled by the King Edward Fund. According to yesterday's papers, King Edward's Fund had not declared whether it was satisfied or not. The Sunday Fund should not be guided by the King Edward Fund, but should refer the matter back to the Council or the Distribution Committee, and the whole grant should drop to the ground without further discussion unless King Edward's Fund declared itself publicly as satisfied. And if it did there should be a general meeting called for the purpose of considering whether the constituents were satisfied.

Mr. Charles Newton Scott (a delegate from Mr. Lewis's parish, St. Mark's, Whitechapel) seconded the amendment.

Sir Savile Crossley complained that the Rev. Mr. Lewis quoted only a portion of the Report of Sir Edward Fry's Committee, a procedure which was always dangerous. He would read another part: "That it is considered right that many specific laboratory examinations, solely for the purpose of helping physicians and surgeons to form correct diagnoses in the exclusive interests of the patients, must be conducted, and that these would have to be paid for by the hospitals in any case." In other words, certain work was done by the medical school which, in hospitals which had no medical schools, would have to be paid for and sent to be done outside the hospital.

Mr. Lewis asked, amid some interruption, whether that work covered the whole of the £1,576, and was called to order by the Chairman, who told him that he was not entitled to cross-examine every speaker who attempted to meet his arguments. If those answers demolished Mr. Lewis's contentions, that gentleman should accept them with good grace. The Chairman was about to put the amendment when

Mr. Edward Counsel pleaded for an opportunity to speak. He said the point submitted by our present King, when Prince of Wales, to the Commission to consider and report upon was whether any, and if any, how much, money given or subscribed for the relief of the sick poor to the twelve London hospitals having medical schools, was contributed directly or indirectly by those hospitals, or any of them, for the maintenance of medical education. The recommendation applied not only to the King's Fund, but to any other source of income, which surely included the Hospital Sunday Fund. As a member of the public, he was under the impression that the matter had been settled once for all, and that money allocated to a medical school must come out of funds specially subscribed.

The Chairman remarked that Sir William Church pointed out that such moneys for the medical school came out of a Discretionary Fund.

Mr. Counsel retorted that a hospital was bound by its own statements issued to the public, and from those it was clear that the money was not out of a Discretionary Fund at all.

Mr. Stow (Parish of St. Jude, Whitechapel) said the question was a critical and serious one, but there was nothing more in it than a serious indictment against St. George's Hospital. He did not agree that the matter could be swept aside in the easy manner which had been suggested; they were at the meeting as trustees of those who had contributed to the Fund, and representing the poor people who had been watching the interesting struggle which had been going on for some time in the papers. He did not put it higher than a grave indictment against St. George's Hospital because the charge had been brought against that hospital for some years. An article on the question by Mr. Stephen Coleridge in the *Contemporary Review* had never been replied to. He fully agreed that the Hospital Sunday Fund was not bound by any other Fund, but those present were trustees of the people, and it would be serious if they did not take a similar line to that of the King Edward Fund with regard to the hospital under discussion. He did not take the view that it reflected at all on the gentlemen who had been elected to administer the Fund. He asked nobody present to do more than suspend judgment on the matter, and it would be a grave dereliction of duty to pass the original resolution under the present circumstances.

Mr. Gerald Fitzgibbon averred that all present had the welfare of the poor people at heart. The amendment meant inquiry, and no hospital should fear inquiry. If St. George's Hospital was doing the right thing it could not object to inquiry. Even though the money now granted may have been expended, and the amendment might consequently seem ridiculous, he urged his hearers to support it, so that every effort could be made to ascertain whether money had been diverted. Either Sir William Church was wrong or the report was wrong, and perhaps Sir William would reply on the point.

The Rev. C. H. Grundy appealed to Mr. Lewis to withdraw his amendment, having succeeded in ventilating the subject. The Council was very vigilant on the matter, and would be glad, before next August, to take any precautions which the Rev. Mr. Lewis might wish. It was desirable, in the interests of the poor, to be as unanimous as possible, as the Fund could not successfully stand those shocks against it.

The Rev. L. S. Lewis said he would be willing to withdraw his amendment on the understanding that the meeting, instead of being held in December, should take place before the grants were made; then the constituents would have a real voice in the affairs of the Fund.

The Chairman said that was impossible, as the Fund was governed by a Constitution, and the Council of the Fund could not enter into any bargain.

The Rev. Mr. Lewis suggested that the constituents should be called together to consider whether they should meet before the grants were made.

The Chairman repeated that the Council could make no such bargain, and proceeded to put the amendment, when eighteen voted for it and thirty-eight against. The original motion was then put and carried.

### Surgical Appliances and other Business.

The Rev. F. W. G. Gilby proposed: "That the Council for the year 1910 be re-elected for 1911, with the addition of the Rev. W. S. Swayne to fill a vacancy." He said he spoke for those who were deaf and dumb, who had received at all hospitals every attention.

The Rev. J. W. P. Chapman seconded, and it was carried.

Sir Joseph Dimsdale proposed: "That June 18 be fixed for Hospital Sunday 1911, and that the cordial cooperation of all ministers of religion within the Metropolitan area be again invited in the usual way." He was sure that all present would desire to convey their sincere gratitude to those ministers of religion who constituted in their several army corps the Church militant on earth. He saw that the subscriptions from the congregations amounted to £40,778, as against £39,143 for the year before.

Mr. Parker Young seconded the motion, and expressed the hope that next year would be a more successful one than the last.

It was unanimously carried.

Mr. J. H. Hale proposed : "That the recommendation of the Council to increase the percentage of the Fund allocated to the purchase of surgical appliances from 5 to 7½ be, and is hereby, adopted, and that Law XI. of the Constitution be altered accordingly. He was sure the constituents had no idea how numerous were the applications for surgical appliances, and 5 per cent. was not by any means adequate to comply with them.

The Rev. C. H. Grundy, in seconding, said his parish had grateful knowledge of the operations of the Fund in this regard.

The resolution was carried without dissent.

Sir E. Durning Lawrence moved a vote of cordial thanks to the Lord Mayor for presiding, which was carried by acclamation.

